# Study on CO_2_ Capture Characteristics and Kinetics of Modified Potassium-Based Adsorbents

**DOI:** 10.3390/ma13040877

**Published:** 2020-02-15

**Authors:** Baihe Guo, Yanlin Wang, Xin Shen, Xiaolei Qiao, Li Jia, Jun Xiang, Yan Jin

**Affiliations:** 1 College of Electrical and Power Engineering, Taiyuan University of Technology, Taiyuan 030024, China; guobaihehappy@163.com (B.G.); 13453122526@163.com (Y.W.); shenshimingxia@outlook.com (X.S.); 13466811581@163.com (X.Q.); 18734869558@163.com (L.J.); 2 State Key Laboratory of Coal Combustion, Huazhong University of Science and Technology, Wuhan 430074, China; Xiangjun@mail.hust.edu.cn

**Keywords:** aerogel, potassium-based adsorbent, modification, CO_2_ capture, kinetics

## Abstract

In this paper, a silica aerogel support was prepared by two-step sol–gel method, and the active component K_2_CO_3_ was supported on the support by wet loading to obtain a modified potassium-based CO_2_ adsorbent. As the influences of reaction conditions on the CO_2_ capture characteristics of modified potassium-based adsorbents, the reaction temperature (50 °C, 60 °C, 70 °C, 80 °C), water vapor concentration (10%, 15%, 20%), CO_2_ concentration (5%, 10%, 12.5%, 15%), and total gas flow rate (400 mL/min, 500 mL/min, 600 mL/min) were studied in a self-designed fixed-bed reactor. At the same time, the low-temperature nitrogen adsorption experiment, scanning electron microscope, and X-ray diffractometer were used to study the microscopic characteristics of modified potassium-based adsorbents before and after the reaction. The results show that the silica aerogel prepared by the two-step sol–gel method has an excellent microstructure, and its specific surface area and specific pore volume are as high as 838.9 m^2^/g and 0.85 cm^3^/g, respectively. The microstructure of K_2_CO_3_ loaded on the support is improved, which promotes the CO_2_ adsorption performance of potassium-based adsorbents. The adsorption of CO_2_ by potassium-based adsorbents can be better described by the Avrami fractional kinetic model and the modified Avrami fractional kinetic model, and it is a complex multi-path adsorption process, which is related to the adsorption site and activity. The optimal adsorption temperature, water vapor concentration, CO_2_ concentration, and total gas volume were 60 °C, 15%, 12.5%, and 500 mL/min, respectively.

## 1. Introduction

As a greenhouse gas, CO_2_ is directly related to global warming. Coal-fired power plants are one of the main sources of CO_2_ emissions. Therefore, the capture, storage, and utilization of CO_2_ in coal-fired power plants have important practical significance for mitigating global warming [1,2,3]. The current CO_2_ emission reduction technology routes of coal-fired power plants mainly are: integrated gas gasification combined cycle (IGCC) technology [4], oxygen-rich combustion system [5,6], chemical chain combustion technology [7,8], physical adsorption [9,10], and chemical absorption method [11]. For existing traditional coal-fired power plants, physical adsorption and chemical absorption methods are more practical methods because they do not require much change in the layout of the power plants. The alkali metal adsorbent combines the process characteristics of physical adsorption method and chemical absorption method, which not only overcomes the shortcomings of low adsorption capacity and poor selectivity to CO_2_ of physical adsorption method, but also eliminates the equipment corrosion problem of chemical absorption method. At the same time, it can react with CO_2_ at low temperature (60–80 °C) and can achieve complete regeneration, and it has small secondary pollution and good cycle performance, so it has received widespread attention [12,13,14,15].

However, this method also has serious disadvantages, such as slow carbonation reaction rate, low conversion rate, and excessive energy consumption for regeneration of the adsorbent. In order to improve the adsorption efficiency, some scholars have supported K_2_CO_3_ on the surface of different supports such as Al_2_O_3_ [16], activated carbon (AC) [17], and 5A molecular sieve [18] to prepare supported potassium-based adsorbents. The experimental research by thermogravimetry found that under the condition of 1% CO_2_ concentration, when the load of K_2_CO_3_ adsorbent supported on Al_2_O_3_ support was increased from 12.8% to 36.8%, the adsorption rate of the adsorbent was increased by 62%. However, the above research used a thermogravimetric analyzer, and the mass of the adsorbent used in the carbonation reaction is relatively small, which was quite different from the actual situation of the power plant.

Studies have shown that the CO_2_ adsorption performance of potassium-based adsorbents mainly depends on the microstructure of the support. Aerogel has a large specific surface area and a high porosity with a regular pore structure, which has a high load capacity and good load characteristics for K_2_CO_3_. Low-cost silica aerogel materials with high porosity and high surface area had been widely used as supports for the synthesis of solid carbon dioxide adsorbents [18,19,20,21], but their active components diethylene triamine (DETA), 3-Aminopropyltrimethoxysilane (APS), and triethylenetetramine (TETA) had poor stability and toxicity. They were easily lost in the flue gas flow and during the carbonation reaction, causing secondary pollution.

Recently, some scholars [22,23] used K_2_CO_3_ solution to impregnate wet gel to prepare silica aerogel-supported K_2_CO_3_ adsorbent. When the design load was 20%, CO_2_ adsorption amount was 1.32 mmol/g, and carbonation conversion rate was 88.62%. However, previous studies had focused on low-concentration CO_2_ capture at room temperature, and the conclusions of the study were not suitable for emission reduction of coal-fired power plants. Based on the previous research by scholars, this article studies the CO_2_ capture characteristics of the adsorbent under the reaction conditions which concentration of CO_2_ from 5% to 15%, water vapor concentration from 10% to 20%, and the reaction temperature from 50 °C to 80 °C.

Adsorption kinetics is an important factor in adsorption characteristics, which directly affects the adsorption efficiency of adsorbents for CO_2_. At present, the adsorption kinetics of amine-based CO_2_ adsorbents has been thoroughly studied [24]. However, there are few studies on the adsorption kinetics of potassium-based adsorbents. At the same time, the adsorption models used in the research are relatively few, and the mechanism of adsorption is not fully explained.

Based on these, in this paper, a two-step sol–gel method was used to prepare silica aerogel support then K_2_CO_3_/silica aerogel modified potassium-based adsorbent was obtained by wet loading. Combined with the microscopic characteristics of modified potassium-based adsorbents, a self-designed fixed-bed reactor was used to study the adsorption reaction characteristics of the adsorbents. Based on the experiments, different adsorption kinetic models were used to fit the experimental data, the model suitable for describing the adsorption process of CO_2_ on the modified potassium-based adsorbent was selected, and the adsorption kinetics was studied.

## 2. Materials and Methods

### 2.1. Experimental Materials and Preparation

#### 2.1.1. Ingredients

For the preparation of highly efficient solid CO_2_ adsorbents, the drugs used were as follows:

Potassium carbonate (K_2_CO_3_, Bodi Chemical Company Limited, Tian Jin, China), tetraethyl orthosilicate (TEOS, Tianli Chemical Reagent Company Limited, Tian Jin, China), absolute ethanol (EtOH, Beichen Founder Reagent Factory, Tian Jin, China), 36.5% hydrochloric acid (Sinopharm Chemical Reagent Company Limited, Shanghai, China), ammonium hydroxide (Kaitong Chemical Reagent Company Limited, Tian Jin, China).

#### 2.1.2. Preparation of Aerogel Support

K_2_CO_3_ was used as the active component, tetraethyl orthosilicate (TEOS) was chosen as the precursor of the silica gel support. The two-step sol–gel wet loading method was used to prepare the adsorbent.

TEOS reacts with water and it is hydrolyzed. The hydrolysis reaction is

(1) $$\mathrm{Si}{\left({\mathrm{OC}}_{2}H_{5}\right)}_{4}+4H_{2}O=\mathrm{Si}{\left(\mathrm{OH}\right)}_{4}+4C_{2}H_{5}\mathrm{OH}$$

The condensation reaction is

(2) $$\mathrm{Si}{\left(\mathrm{OH}\right)}_{4}+\mathrm{Si}{\left(\mathrm{OH}\right)}_{4}={\left(\mathrm{OH}\right)}_{3}\mathrm{Si}-O-\mathrm{Si}{\left(\mathrm{OH}\right)}_{3}+H_{2}O$$

(3) $$\mathrm{Si}{\left({\mathrm{OC}}_{2}H_{5}\right)}_{4}+\mathrm{Si}{\left(\mathrm{OH}\right)}_{4}={\left(\mathrm{OH}\right)}_{3}\mathrm{Si}-O-\mathrm{Si}{\left(\mathrm{OH}\right)}_{3}+C_{2}H_{5}\mathrm{OH}$$

The polymerization reaction is

(4) $$n\left(\mathrm{Si}-O-\mathrm{Si}\right)={\left(-\mathrm{Si}-O-\mathrm{Si}-\right)}_{n}$$

The total reaction of hydrolysis, condensation and polycondensation is

(5) $$\mathrm{Si}{\left({\mathrm{OC}}_{2}H_{5}\right)}_{4}+2H_{2}O={\mathrm{SiO}}_{2}+4C_{2}H_{5}\mathrm{OH}$$

Completely hydrolyzing 1 mol of TEOS requires 2 mols of H_2_O. Properly increasing the ratio of H_2_O can promote the reaction. Ethanol is solvent for TEOS to react with H_2_O. HCl is used to adjust pH value. Under acidic condition, TEOS is prone to hydrolysis.

The operation steps were as follows: TEOS, water, absolute ethanol, and 36.5% hydrochloric acid were mixed in a molar ratio of 1:3:15:0.001. The mixture was stirred for 1 h using a magnetic stirrer (Guang Ming, Bei Jing, China) under heating in a 50 °C water bath. After cooling to room temperature, ammonia water was added dropwise according to the ratio of TEOS: ammonia water = 1:0.002. Alkaline condition helps the polycondensation reaction to occur, so ammonia water is added to change the pH value. Then stirred the mixture at room temperature for 1 min using a magnetic stirrer to form a sol. After stewing for 2 h, the beaker was tilted at 45° without liquid flowing out meant a wet gel was formed. The wet gel was aged for 48 h, then placed in an oven (Gang Yuan, Tian Jin, China) dried at 120 °C. The dried gel was placed in a muffle furnace (Ke Jing, Zheng Zhou, China) and calcined at 300 °C for 2 h to obtain a silica gel support.

#### 2.1.3. K_2_CO_3_ Loading into Aerogel Support

K_2_CO_3_ was dissolved in an aqueous solution of ethanol at a 25% theoretical load, and a certain amount of carrier was weighed and added. It was stirred at room temperature for 12 h using a magnetic stirrer and then placed in an oven for drying. In order to avoid changing the active components, no calcination was carried out after drying. Finally, it was ground and sieved to obtain the adsorbent.

### 2.2. Experimental Methods

**Figure 1:**
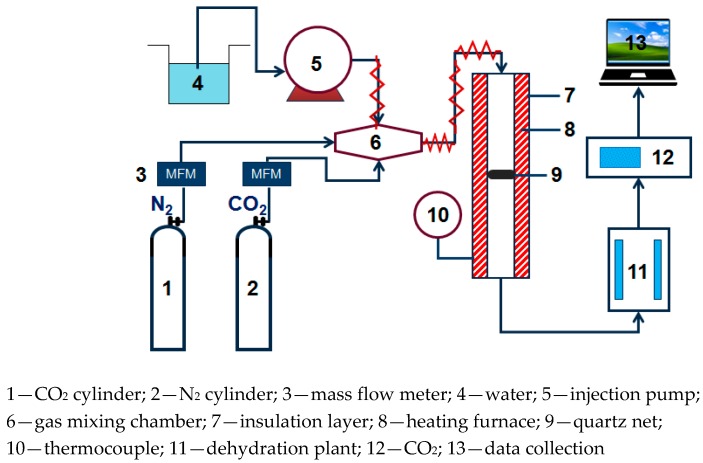
Fixed bed reaction device diagram.

The small fixed bed experimental system used in CO_2_ adsorption experiment is shown in Figure 1, N_2_ and CO_2_ are supplied through a gas cylinder. The water vapor is generated by electric heating and metering controlled by the Series III metering pump (SSI, Cincinnati, OH, USA). N_2_, CO_2_, and water vapor are mixed in the gas mixing chamber. After passing into the dehydration device, the exhaust gas of the adsorption reaction is dried and dehydrated, then connected to an S2000 CO_2_ gas analyzer (Xin Ze, Shan Dong, China) to perform on-line monitoring of the concentration.

In the adsorption experiment, the mass of the modified potassium-based adsorbent used was 2 g. In order to simulate the actual flue gas environment of the power plant, the basic experimental reaction atmosphere was: 10% CO_2_ concentration, 10% water vapor concentration, and the rest was N_2_. The parameters, reaction temperature (50 °C, 60 °C, 70 °C, 80 °C), water vapor concentration (10%, 15%, 20%), CO_2_ concentration (5%, 10%, 12.5%, 15%), and total gas volume (400 mL/min, 500 mL/min, 600 mL/min) were changed to obtain the influence of operating conditions on the adsorption reaction characteristics.

The decarburization performance of the adsorbent was studied by the CO_2_ adsorption breakthrough rate. The calculation method is shown in Equation (6).

(6) $$\eta =\frac{c_{\mathrm{out}}}{c_{\mathrm{in}}}$$

In the equation, η is CO_2_ adsorption breakthrough rate, %; $c_{\mathrm{in}}$ is CO_2_ concentration in the mixed gas before the adsorption reaction, %; $c_{\mathrm{out}}$ is CO_2_concentration in the mixed gas after the adsorption reaction, %.

The adsorption capacity of the adsorbent was calculated by the amount of CO_2_ (mmol/g) adsorbed by the potassium-based adsorbent per unit mass, and the calculation method is shown in Equation (7).

(7) $$q=\int_{0}^{t}\frac{c_{\mathrm{in}}-c_{\mathrm{out}}}{1-c_{\mathrm{out}}}\mathrm{Qdt}\times \frac{1}{22400m}\times \frac{T}{T_{0}}$$

In the equation, q is CO_2_ cumulative adsorption amount, mmol/g; Q is total gas flow rate, mL/min; m is the mass of the adsorbent before the reaction, g; T is the temperature of the adsorption reaction, K; T_0_ is 273 K.

In this paper, JEOL JSM-7800F (Japan Electronics Corporation, Tokyo, Japan) ultra-high-resolution field emission scanning electron microscope was used to observed the microscopic morphology of the adsorbent surface. ASAP 2460 nitrogen analyzer (Micromeritics, Norcross, GA, USA) was used for N_2_ adsorption and desorption experiments, and the specific surface area was calculated using BET method. BJH method was used to obtain the pore structure parameters. DX-2700 X-ray diffractometer (Fang Yuan Instrument Company Limited, Dandong, China) was used to obtain the crystal structure characteristics of the adsorbent before and after the reaction. Epsilon1 scientific research X-ray fluorescence spectrometer (PANalytical B.V., Almelo, the Netherlands) was used to detect the experimental loading of each component in the prepared adsorbent.

### 2.3. Kinetic Models

The intrinsic reaction of potassium-based adsorbent for CO_2_ adsorption is the chemical reaction of K_2_CO_3_ with CO_2_ and H_2_O, which is a non-catalytic heterogeneous gas–solid reaction. It mainly includes the following basic processes: CO_2_ and H_2_O diffuse into the surface and pores of the modified potassium-based adsorbent; CO_2_ and H_2_O react with the active sites of modified potassium-based adsorbents; a dense product layer is formed. As the reaction proceeds, the product layer becomes thicker, and intra-particle diffusion becomes difficult. There are many kinetic models used to describe the adsorption kinetic properties on solid adsorbents currently. Among these models, pseudo-first order and pseudo-second order kinetic models have been widely used to represent the gas–solid adsorption process [25,26,27]. Among them, the pseudo-first order kinetic model, as shown in Equation (8), mainly studies the adsorption process controlled by surface diffusion. Pseudo-second order kinetic model, as shown in Equation (9), based on the Langmuir adsorption isotherm equation, a hypothesis is established that the chemical reaction is the rate-controlling step of the adsorption process on the gas–solid interface. It mainly describes the chemical adsorption process and the formation of chemical bonds. Both the pseudo-first order kinetic model and the pseudo-second order kinetic model are applicable to gas–solid adsorption reactions on porous adsorbents. Although they can be used to obtain kinetic parameters of the adsorption process, the gas–solid adsorption process of modified potassium-based adsorbents may involve multiple processes and is more complicated. These two models may have some limitations. The Weber–Morris kinetic model [28] mainly describes the diffusion process of substances in the internal pores of the particles during the solid adsorption process. It is not suitable for surface diffusion controlled reactions, as shown in Equation (10). The Elovich kinetic model [29] is based on the Temkin adsorption isotherm equation and describes a series of reaction mechanism processes, including surface diffusion, internal diffusion, activation, and deactivation, etc. It is suitable for processes with large changes in activation energy, such as the Equation (11) shows. The Avrami fractional kinetic model is a crystallization kinetic model, which is suitable for describing the process of random nucleation and subsequent growth. Rodrigo Serna-Guerrero [30] used the Avrami fractional kinetic model to describe the kinetics of CO_2_ adsorption by amine-functionalized mesoporous silica gel, as shown in Equation (12). Aliakbar Heydari-Gorji [31] proposed a modified Avrami fractional kinetic model. The model already includes multiple adsorption pathways, including surface diffusion, intra-particle diffusion, and interaction with active sites (physical and chemical). It was used to describe the CO_2_ adsorption kinetics of an adsorbent with an amine active site, as shown in Equation (13).

The kinetic models are as follows:

1. Pseudo-first order kinetic model

(8) $$q=q_{e}\left(1-e^{-{\mathrm{tk}}_{1}}\right)$$

In the equation, q is adsorption amount per unit mass of adsorbent, mmol/g; q_e_ is adsorption amount per unit mass of adsorbent at equilibrium, mmol/g; t is adsorption time, min; k_1_ is pseudo-first order rate constant, min^−1^.

2. Pseudo-second order kinetic model

(9) $$q=\frac{q_{e}^{2}k_{2}t}{1+q_{e}k_{2}t}$$

In the equation, k_2_ is pseudo-second order rate constant, mmol/(g·min).

3. Weber–Morris kinetic model

(10) $$q=k_{\mathrm{id}}t^{1/2}+C$$

In the equation, k_WB_ is Weber–Morris diffusion rate constant, g/(g·min^1/2^); C is constant related to boundary layer thickness.

4. Elovich kinetic model

(11) $$q=\frac{1}{\beta }\ln\left(t+t_{0}\right)-\frac{1}{\beta }\ln\left(t_{0}\right)$$

In the equation, k_E_ is initial adsorption rate, g/(g·min^1/2^); β is constant related to surface coverage and activation energy; t_0_=1/(k_E_·β).

5. Elovich kinetic model

(12) $$q=q_{e}\left(1-e^{-\frac{{\left(k_{a}t\right)}^{n}}{n}}\right)$$

In the equation, k_a_ is Avrami rate constant, min^−1^; n is Avrami exponent.

6. Modified Avrami fraction kinetic model

(13) $$q=q_{e}-\frac{1}{{\left[\left(n-1\right)\frac{k_{\mathrm{ma}}}{m}t^{m}+\frac{1}{q_{e}{}^{n-1}}\right]}^{\frac{1}{n-1}}}$$

In the equation, k_ma_ is modified Avrami rate constant, g^n−1^min^−m^mmol^1−n^; m,n are modified Avrami exponent.

## 3. Results and Discussion

### 3.1. Adsorbent Characterization

The experimental loading of the active component K_2_CO_3_ in the modified potassium-based adsorbent was detected by XRF. The designed loading and experimental loading are 25% and 18.21% respectively, as shown in the Table 1.

In order to study the influence of the microstructure of the modified potassium-based adsorbent on CO_2_ adsorption performance, low-temperature N_2_ adsorption, and desorption experiments were performed on the adsorbent, and the BET specific surface area and BJH cumulative pore volume were calculated.

Table 2 shows the results of low-temperature N_2_ adsorption/desorption experiments on modified potassium-based adsorbents. It can be seen from Table 2 that BET specific surface area and cumulative pore volume of K_2_CO_3_ are small and the microstructure is poor. The adsorption of CO_2_ by K_2_CO_3_ is mainly chemical adsorption. The microstructure of silica aerogel as support is well developed, and BET specific surface area and cumulative pore volume are 838.94 m^2^/g and 0.8482 cm^3^/g, respectively. The microstructure of the modified potassium-based adsorbent prepared by loading K_2_CO_3_ on silica aerogel is greatly improved. Both BET specific surface area and cumulative pore volume are greatly increased, which is conducive to the full contact of K_2_CO_3_ and CO_2_, and then enhances the adsorption of CO_2_ by potassium-based adsorbents. It can be known from the pore size distribution that the pores of the silica aerogel support are mainly mesopores with a small number of micropores. The modified potassium-based adsorbent after loading is mainly mesopores with very few large pores, the relative specific pore volume of mesopore is 98.95%. Mesopores are beneficial to the diffusion and adsorption reaction of CO_2_ in the pores.

**Figure 2:**
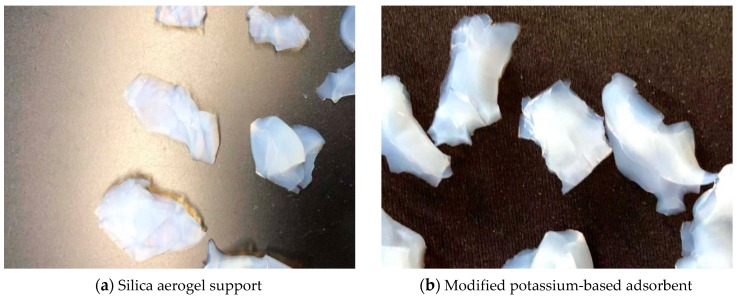
Actual images of support and adsorbent.

Figure 2 is a set of actual images of aerogel support prepared by two-step sol–gel method and modified potassium-based adsorbent obtained after loading. Figure 2a is aerogel support, showing a translucent light blue; Figure 2b is modified potassium-based adsorbent, showing an opaque white.

**Figure 3:**
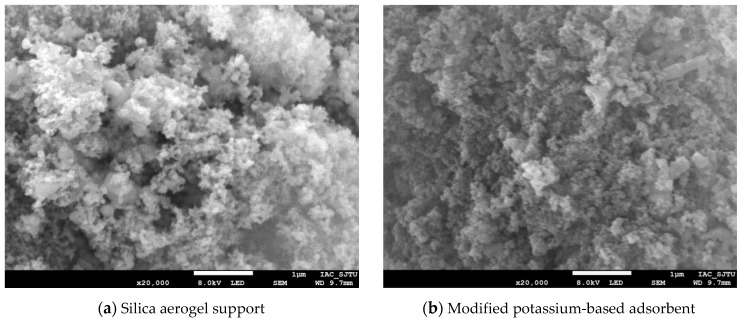
SEM image of support and adsorbent.

The silica aerogel support and modified potassium-based adsorbent were observed by scanning electron microscope (SEM), and the microscopic characteristics and surface morphology of the silica aerogel support and modified potassium-based adsorbent were obtained, as shown in Figure 3. As can be seen from Figure 3a, the surface of the silica aerogel support has loose structure with lots of pores. After the active component of K_2_CO_3_ loading, the pores are filled with a large amount of K_2_CO_3_ and there are still obvious pores between the particles, as Figure 3b shows.

**Figure 4:**
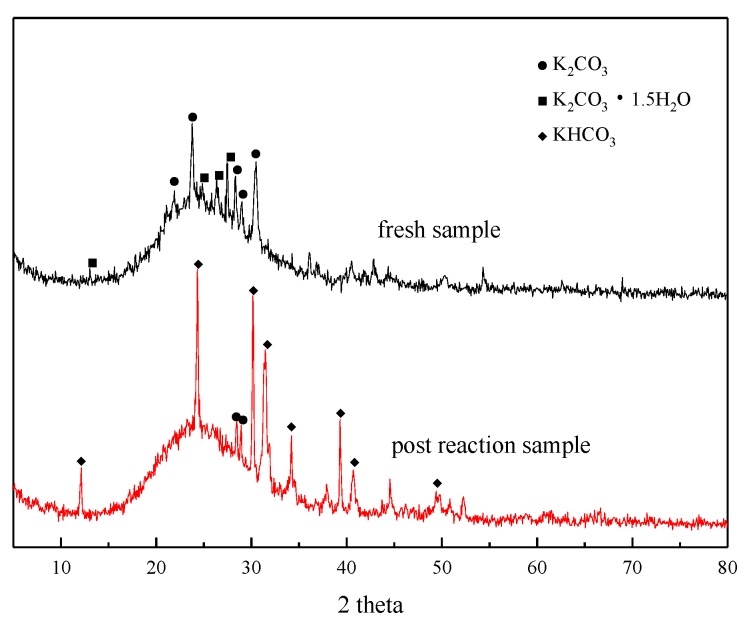
XRD patterns before reaction and after reaction.

XRD analysis was performed before and after the adsorption reaction of the modified potassium-based adsorbent to obtain the change in the composition of the adsorbent during the adsorption process. The results are shown in Figure 4. It can be seen from the figure that a bun-shaped peak appears between 18° and 35°, which is due to the silica gel support containing an amorphous phase in the adsorbent. There is a large amount of K_2_CO_3_ and K_2_CO_3_·1.5H_2_O in the adsorbent before the reaction. KHCO_3_ is formed after the reaction, and unreacted K_2_CO_3_ can be detected at the same time.

### 3.2. Comparison of Kinetic Models

**Figure 5:**
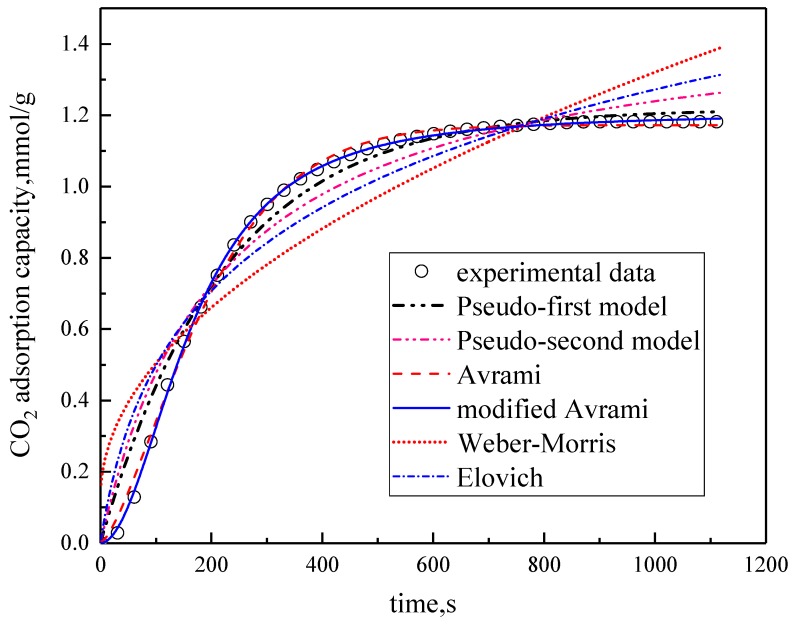
Fitting results for experimental data of six models.

Under the conditions of the temperature was 60 °C, the water vapor concentration was 10%, CO_2_ concentration was 5%, and the total gas volume was 550 mL/min, experimental data fitting was performed on several kinetic models. The results are shown in Figure 5 and Table 3. Among them, the correlation coefficients of pseudo-first order kinetic model, pseudo-second order kinetic model, Avrami fractional kinetic model and modified Avrami fractional kinetic model are high, and the modified Avrami fractional kinetic model shows the highest correlation coefficient. Weber–Morris kinetic model and Elovich kinetic model have poor fitting results, which are not suitable for describing the adsorption of modified potassium-based adsorbents on CO_2_. The experimental results are consistent with the pseudo-first order and pseudo–second order kinetic models, indicating that the adsorption rate of modified potassium-based adsorbents for CO_2_ is controlled by both surface diffusion and chemical reactions at the gas–solid interface. The results mean that the adsorption reaction included both physical adsorption and chemical adsorption. The experimental fitting results are consistent with Avrami fractional kinetic model and the modified Avrami fractional kinetic model, indicating that the CO_2_ adsorption on modified potassium-based adsorbents is not a single process, but a complex multi-path adsorption process, which is related to the adsorption site and activity. The correlation coefficient of Avrami fractional kinetic model is 0.9977, and the correlation coefficient of the modified Avrami fractional kinetic model is 0.9998. These two models were selected as the adsorption kinetics model of the modified potassium-based adsorbent.

### 3.3. Effects of Reaction Conditions on Adsorption Characteristics

In order to obtain the influence of different reaction conditions on the adsorption characteristics, the adsorption reaction experiment was carried out under different conditions of temperature, water vapor concentration, CO_2_ concentration, and total gas volume. At the same time, performed the Avrami fraction kinetic model and the modified Avrami fraction kinetics model fitting calculations on experimental data.

#### 3.3.1. Reaction Temperature

**Figure 6:**
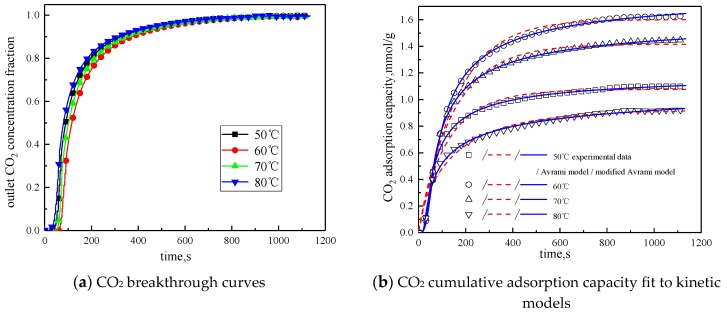
Adsorption characteristics and model fitting results at different temperatures.

The concentration of water vapor was 15 %, the concentration of CO_2_ was 12.5%, the rest was N_2_, and the total gas volume was 550 mL/min. Adsorption experiments were performed on modified potassium-based adsorbent under different reaction temperature conditions (50 °C, 60 °C, 70 °C, 80 °C). The results are shown in Figure 6 and Table 4.

It can be known from Table 4 that with the increase of the reaction temperature, the reaction rates k_a_ and k_ma_ obtained by fitting Avrami fractional kinetic model and the improved Avrami fractional kinetic model increase, indicating that the increase in temperature make the kinetic parameters increase. According to Figure 6, the reaction temperature increases from 50 °C to 80 °C, with the reaction temperature increases, the cumulative adsorption amount increases first and then decreases, and the optimal reaction temperature is 60 °C. The reaction temperature increases from 50 °C to 60 °C, the amount of CO_2_ adsorption increases. This is because the increase of temperature can promote chemical bond break, enhance the activity of the active site of the modified potassium-based adsorbent, resulting in chemical adsorption increasing. At the same time, the increase of temperature promotes the diffusion of CO_2_ on the surface and internal pores of the modified potassium-based adsorbent, which is beneficial to the CO_2_ adsorption reaction. The reaction temperature increases from 60 °C to 80 °C, the amount of CO_2_ adsorption decreased. The reason for this phenomenon is that the chemical reaction of the potassium-based adsorbent to adsorb CO_2_ is a reversible reaction. The process of adsorbing CO_2_ and generating KHCO_3_ at the same time is an exothermic process. The temperature above 60 °C is not conducive to the forward direction of the reaction, leading to a reduction in the amount of CO_2_ adsorption. The literature [16] suggested that, if the temperature was higher than 90 °C, the forward reaction of K_2_CO_3_ and CO_2_ did not occur.

Adsorption activation energy refers to the energy required for adsorbate molecules to become activated before adsorption. The adsorption activation energy can be obtained by the Arrhenius equation, as shown in Equation (14).

(14) $$\mathrm{lnk}=-\frac{E_{a}}{\mathrm{RT}}+{\mathrm{lnk}}_{0}$$

In the equation, E_a_ is adsorption activation energy, kJ/mol; R is gas constant, 8.314 J/(mol·K); k_0_ is temperature effect factor.

**Figure 7:**
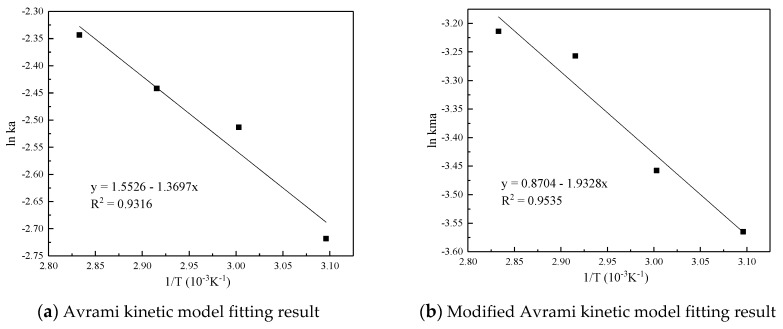
Linear fitting of the CO_2_ adsorption on the adsorbent by Arrhenius equation.

Figure 7 and Table 5 are the results and fitting parameters calculated from the linear correlation regression of the experimental data of Avrami fractional kinetic model and the modified Avrami fractional kinetic model using the Arrhenius equation. The activation energy is 11.3877 kJ/mol and 16.0697 kJ/mol, respectively. Because the modified Avrami model considers the complex mechanisms of multiple response pathways, and the correlation coefficient is higher, the calculation result of the modified Avrami model is considered to be more accurate.

#### 3.3.2. Water Vapor Concentration

**Figure 8:**
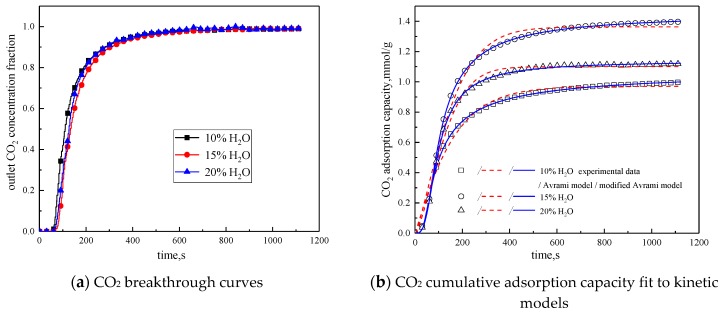
Adsorption characteristics and model fitting results with different water vapor concentrations.

In order to study the influence of water vapor concentration on the adsorption characteristics of modified potassium-based adsorbents, the experiments were conducted with water vapor concentrations of 10%, 15%, and 20%. The reaction temperature was 60 °C, CO_2_ concentration was 10 %, and the total gas volume was 550 mL/min. The experimental results are shown in Figure 8 and Table 6.

From the experimental results, it can be seen that as the water vapor concentration increases, the reaction rates k_a_ and k_ma_ obtained from the kinetic model fitting increase, and water vapor positively promotes the kinetic parameters. From the penetration curve and cumulative adsorption curve, the optimal water vapor concentration is 15%. The water vapor concentration increases from 10 % to 15 %, and the cumulative adsorption amount increases. When K_2_CO_3_ chemically reacts with CO_2_ in the presence of water vapor, reaction (15) occurs first, and the active site K_2_CO_3_·1.5H_2_O is formed. As the water vapor concentration increases, reaction (15) can be promoted to generate more K_2_CO_3_·1.5H_2_O. Then reaction (16) occurs, the active site K_2_CO_3_·1.5H_2_O reacts with CO_2_ to form K_4_H_2_(CO_3_)_3_·1.5H_2_O, which is unstable and easily reacts with CO_2_ continuously. Following, reaction (17) occurs to produce KHCO_3_. Therefore, the increase of water vapor concentration promotes the occurrence of the reaction and increases the amount of adsorption. The water vapor concentration increases from 15% to 20%, and the cumulative adsorption amount decreases. This is because the excess water vapor forms a liquid film and causes the agglomeration of the modified potassium-based adsorbent, causing the plugging of the pore structure. CO_2_ cannot be brought into contact with the active site smoothly, and eventually the cumulative adsorption amount is reduced.

(15) $$K_{2}{\mathrm{CO}}_{3}\left(s\right)+1.5H_{2}O\left(g\right)↔K_{2}{\mathrm{CO}}_{3}\cdot 1.5H_{2}O\left(s\right)$$

(16) $$2K_{2}{\mathrm{CO}}_{3}\cdot 1.5H_{2}O\left(s\right)+{\mathrm{CO}}_{2}\left(g\right)↔K_{4}H_{2}{\left({\mathrm{CO}}_{3}\right)}_{3}\cdot 1.5H_{2}O\left(s\right)+0.5H_{2}O\left(g\right)$$

(17) $$K_{4}H_{2}{\left({\mathrm{CO}}_{3}\right)}_{3}\cdot 1.5H_{2}O\left(s\right)+{\mathrm{CO}}_{2}\left(g\right)↔4{\mathrm{KHCO}}_{3}\left(s\right)+0.5H_{2}O\left(g\right)$$

#### 3.3.3. CO_2_ Concentration

**Figure 9:**
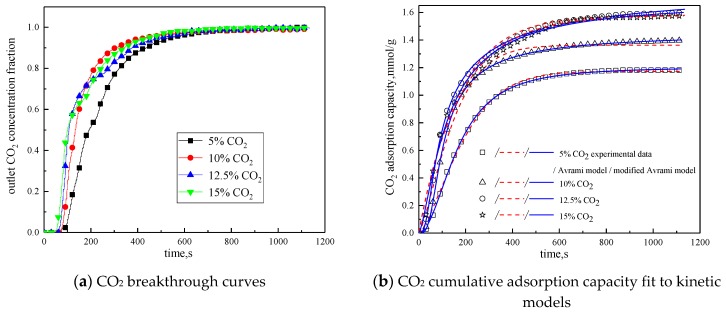
Adsorption characteristics and model fitting results with different CO_2_ concentrations.

Since CO_2_ concentration in the reaction atmosphere had a certain effect on the characteristics of the adsorption reaction, it was changed. The characteristics of the adsorption reaction were studied when CO_2_ concentration was 5 %, 10 %, and 15 %, respectively. Considering the composition of the power plant flue gas, between the CO_2_ concentration of 10 % and 15 %, 12.5 % CO_2_ concentration was added in the reaction atmosphere. The rest was N_2_. Other reaction conditions were reaction temperature of 60 °C, water vapor concentration of 15 %, and total gas volume of 550mL/min. The results of the adsorption reaction experiments are shown in Figure 9 and Table 7.

It can be seen from Figure 9 that when CO_2_ concentration is 5 %, the breakthrough time is the longest, but the cumulative adsorption amount is the lowest; when CO_2_ concentration is increased from 5% to 10%, the breakthrough time is reduced, but the cumulative adsorption amount is increased; CO_2_ concentration increases from 10% to 12.5%, the breakthrough time is further shortened, and the cumulative adsorption amount increases; while CO_2_ concentration increases from 12.5% to 15%, the breakthrough time and cumulative adsorption amount remains basically unchanged. This is because during the reaction, the difference between CO_2_ concentration in the reaction atmosphere and CO_2_ concentration on the surface of the modified potassium-based adsorbent is the driving force for the diffusion of the adsorption reaction surface. Increasing CO_2_ concentration in the reaction atmosphere can increase the adsorption rate and thus promote the adsorption reaction. In addition, the lower CO_2_ concentration is quickly consumed in the adsorption process, so increasing CO_2_ concentration at a low CO_2_ concentration can increase the cumulative adsorption amount. However, under the condition that the number of adsorption sites and the adsorption activity on the surface of the modified potassium-based adsorbent remain unchanged, the increases in CO_2_ concentration causes the difference between CO_2_ concentration on the surface of the modified potassium-based adsorbent and CO_2_ concentration in the reaction atmosphere decreases. When CO_2_ concentration is increased to a certain extent, the difference in concentration will not change, and the external diffusion resistance will increase, so that the amount of adsorbent will not change.

It can be seen from the experimental results that under the conditions of reaction temperature is 60 °C, CO_2_ concentration is 12.5%, water vapor concentration is 15%, and total gas volume is 550 mL/min, the CO_2_ adsorption capacity of the modified potassium-based adsorbent prepared in this paper reaches 1.5979 mmol/g, which is better than that in the literature [18,23,24].

#### 3.3.4. Gas Flow Rate

**Figure 10:**
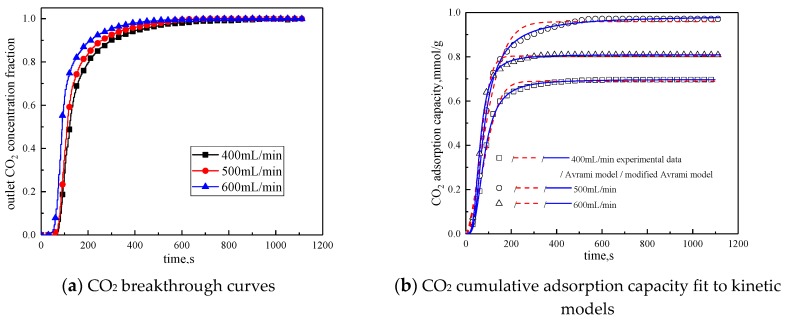
Adsorption characteristics and model fitting results with different gas volumes.

The gas flow rate was changed to study the influence of different gas flow rates on the adsorption characteristics of modified potassium-based adsorbents. The adsorption reaction temperature was 60 °C, the water vapor concentration was 10%, the CO_2_ concentration was 10%, and the rest was N_2_. Adsorption reactions were performed under the condition total gas volume of 400 mL/min, 500 mL/min, and 600 mL/min. The results are shown in Figure 10 and Table 8.

It can be seen from Figure 10 that the total gas volume increases from 400 mL/min to 600 mL/min. With the increase of total gas volume, the breakthrough time gradually decreases, and the cumulative adsorption volume increases first and then decreases. When the total gas volume is 500 mL/min, the cumulative adsorption capacity is the maximum. The reason is when the gas flow rate is low, increasing the gas flow rate can reduce the surface diffusion and internal diffusion mass transfer resistance. So when the gas volume increases from 400 mL/min to 500 mL/min, the cumulative adsorption capacity is increased. The gas flow rate further increases, the high gas velocity will shorten the contact time between the reactant CO_2_ and the active site of the modified potassium-based adsorbent. Therefore, when the gas volume increases from 500 mL/min to 600mL/min, the cumulative adsorption capacity decreased. It can be concluded from Table 8 that the airflow speed increases, the fitting reaction rates k_a_ and k_ma_ increases, so increasing the gas flow rate is beneficial to the reaction rate.

## 4. Conclusions

In this paper, a silica aerogel support was prepared by two-step sol–gel method, and the active component K_2_CO_3_ was supported on the silica aerogel support according to theoretical loading of 25% to obtain a modified potassium-based adsorbent. Combined with the microscopic characteristics of the adsorbent, the self-designed fixed-bed reactor was used to study the adsorption characteristics of the modified potassium-based adsorbent with the help of nitrogen adsorption instrument, scanning electron microscope, and X-ray diffractometer. The kinetic models were fitted to the experimental data. The results show:

(1) The specific surface area and cumulative pore volume of the active component K_2_CO_3_ are small. After loading it on the prepared silica aerogel support, the microstructure of the modified potassium-based adsorbent obtained is improved. The specific surface area is increased to 110.46 m^2^/g, and the cumulative pore volume is 0.0857 cm^3^/g. The mesopores reaches more than 98%. The developed microstructure is favorable for the CO_2_ capture of the adsorbent.

(2) Among the six kinetic models, the Avrami fractional kinetic model and the modified Avrami fractional kinetic model show the highest correlation coefficients with R^2^ more than 0.97. 60 °C is the optimal reaction temperature. The influence of temperature on the adsorption characteristics is both positive and negative. Increasing the temperature can promote the increase of the adsorption rate. According to the characterization results, the reaction of the adsorbent to capture CO_2_ is that K_2_CO_3_ reacts with CO_2_ to generate KHCO_3_. This reaction is reversible and exothermic, and the excessively high temperature results in a decrease in the amount of adsorption.

(3) According to the kinetic studies, increasing the water vapor concentration increases the adsorption rate. When the water vapor concentration is 15%, the cumulative adsorption amount reaches a peak. The increase of the water vapor content can promote the formation of active sites. However, the excess water vapor makes the microstructure of the adsorbent poor and the mass transfer worse, which is not conducive to adsorption.

(4) There is an optimal value of CO_2_ concentration. Increasing the CO_2_ concentration within a certain range can increase the driving force for surface diffusion of the adsorption reaction, thereby increasing the amount of adsorption. After the CO_2_ concentration exceeds 12.5%, the amount of adsorption no longer increases.

(5) Increasing the gas flow rate can increase the adsorption rate as kinetic studies shows, while its effect on the amount of adsorption is two-fold. The lower gas velocity can make the contact time between the reactant CO_2_ and the active site longer, and the higher gas velocity can make the diffusion resistance less. Generally, with the gas velocity increases, the cumulative adsorption capacity increases first and then decreases. The gas flow rate of 500 mL/min is optimal.

The results of this article lay the foundation for the practical application of CO_2_ emission reduction in coal-fired power plants. The next work will study the law and mechanism of the failure of the adsorbent for multiple cycles, and the adsorption performance of the adsorbent in the presence of other acidic gases in the flue gas.

## Figures and Tables

**Table 1:** Experimental loading of adsorbent

Designed Loading	Designed K Content	Experimental K Content	Experimental Loading
25%	14.13%	10.29%	18.21%

**Table 2:** Pore structure parameters of active component, support, and adsorbent.

	BET Specific Surface Area m^2^/g	Cumulative Pore Volume cm^3^/g	Relative Specific Pore Volume %		
			Micropore	Mesopore	Macropore
K_2_CO_3_	2.28	0.0050	1.46	65.01	33.52
Silica aerogel support	838.94	0.8482	8.54	91.40	0.06
Modified adsorbent	110.46	0.0857	0	98.95	1.05

**Table 3:** Fitting parameters for experimental data of six models

Models	Parameter Calculation Results					
Pseudo–first order	SSE%	R^2^	q mmol/g	k_1_ min^−1^		
	2.5372	0.9866	1.2181	0.0448		
Pseudo–second order	SSE%	R^2^	q mmol/g	k_2_ mmol/(g·min)		
	5.7308	0.9605	1.5075	0.0306		
Weber–Morris	SSE%	R^2^	k_WB_ mmol/(g·min^1/2^)	C		
	22.1566	0.8164	0.0377	0.1267		
Elovich	SSE%	R^2^	k_E_ mmol/(g·min^1/2^)	β	t_0_	
	10.454	0.9176	0.3832	2.6095	37.4943	
Avrami	SSE%	R^2^	q mmol/g	k_a_ min^−1^	n	
	0.3128	0.9977	1.1724	0.0602	1.4161	
Modified Avrami	SSE%	R^2^	q mmol/g	k_ma_ g^n−1^min^−m^mmol^1−n^	m	n
	0.0483	0.9998	1.204	0.0196	1.937	0.7586

**Table 4:** Fitting parameters of experimental data under different temperature conditions

t °C		50	60	70	80
Experimental result	q mmol/g	1.0990	1.6283	1.4513	0.9226
Avrami	SSE%	2.2402	3.6465	1.5201	1.0813
	R^2^	0.9820	0.9862	0.9759	0.9750
	q mmol/g	1.4170	1.9525	1.0766	0.9230
	k_a_ min^−1^	0.0667	0.0813	0.0874	0.0960
	n	1.5529	1.5343	1.4824	1.6509
Modified Avrami	SSE%	0.0510	0.1293	0.0617	0.1468
	R^2^	0.9995	0.9994	0.9989	0.9964
	q mmol/g	1.6396	2.2355	1.2131	1.2212
	k_ma_ g^n−1^min^−m^mmol^1−n^	0.0283	0.0315	0.0385	0.0402
	m	1.9583	1.7364	1.9234	1.8759
	n	0.3127	0.2401	0.3168	0.5719

**Table 5:** Arrhenius equation parameters

Models	E_a_ kJ/mol	R^2^
Avrami	11.3877	0.9316
Modified Avrami	16.0697	0.9535

**Table 6:** Fitting parameters of experimental data for adsorption characteristics under different water vapor concentrations

	Water Vapor Concentration	10%	15%	20%
Experimental result	q mmol/g	0.9969	1.3966	1.1189
Avrami	SSE%	0.9713	1.7977	0.9782
	R^2^	0.9855	0.9877	0.9891
	q mmol/g	0.9707	1.3629	1.0999
	k_a_ min^−1^	0.0707	0.0778	0.0948
	n	1.1095	1.3203	1.4804
Modified Avrami	SSE%	0.0169	0.0307	0.0514
	R^2^	0.9997	0.9997	0.9993
	q mmol/g	1.0808	1.4537	1.1379
	k_ma_ g^n−1^min^−m^mmol^1−n^	0.0206	0.0289	0.0498
	m	1.8344	1.9202	1.8419
	n	0.6152	0.9896	0.2135

**Table 7:** Fitting parameters of experimental data for adsorption characteristics under different CO_2_ concentrations

	CO_2_ Concentration	5%	10%	12.5%	15%
Experimental result	q mmol/g	1.1815	1.3966	1.5979	1.5733
Avrami	SSE%	0.3128	1.7977	1.8463	1.1013
	R^2^	0.9977	0.9877	0.9894	0.9932
	q mmol/g	1.1725	1.3629	1.5813	1.5783
	k_a_ min^−1^	0.0603	0.0778	0.0819	0.0845
	n	1.4162	1.3203	1.5156	1.4740
Modified Avrami	SSE%	0.0483	0.0307	0.2801	0.8862
	R^2^	0.9998	0.9997	0.9982	0.9943
	q mmol/g	1.2040	1.4537	1.8253	1.6894
	k_ma_ g^n−1^min^−m^mmol^1−n^	0.0217	0.0289	0.0326	0.0381
	m	1.9370	1.9202	2.0355	1.8372
	n	0.7586	0.9896	0.3425	0.1390

**Table 8:** Fitting parameters of experimental data for different gas adsorption characteristics

	Gas Flow Rate mL/min	400	500	600
Experimental result	q mmol/g	0.6967	0.9711	0.8083
Avrami	SSE%	0.2159	0.2076	0.2453
	R^2^	0.9924	0.9931	0.9921
	q mmol/g	0.6892	0.9584	0.8030
	k_a_ min^−1^	0.0412	0.0637	0.0841
	n	1.4149	1.5550	1.5372
Modified Avrami	SSE%	0.0231	0.0206	0.0339
	R^2^	0.9991	0.9991	0.9989
	q mmol/g	0.6978	0.9858	0.8091
	k_ma_ g^n−1^min^−m^mmol^1−n^	0.0160	0.0176	0.0291
	m	1.8447	1.7000	1.9117
	n	0.4539	0.6902	0.5180
